# Supplementary material for: Trunk kinematics and motor unit behavior during different loads and speeds in individuals with and without aberrant movement patterns during active forward bending: A cross-sectional study
Source: PLoS One. 2025 Apr 16;20(4):e0321084. doi: 10.1371/journal.pone.0321084 (PMC12002531; doi:10.1371/journal.pone.0321084)

PLOS ONE

Trunk kinematics and motor unit behavior during different loads and speeds in individuals with and without aberrant movement patterns during active forward bending: A cross-sectional study

--Manuscript Draft--

Manuscript Number:	PONE-D-24-57098
Article Type:	Research Article
Full Title:	Trunk kinematics and motor unit behavior during different loads and speeds in individuals with and without aberrant movement patterns during active forward bending: A cross-sectional study
Short Title:	Trunk kinematics and motor unit behavior
Corresponding Author:	Peemongkon Wattananon, PhD<br>Mahidol University<br>Salaya, Nakhon Pathom THAILAND
Keywords:	Decomposition electromyography; Instability catch; kinematics; Motor unit behavior; Aberrant movement pattern; Neuromuscular control
Abstract:	Background<br><br>Instability catch (IC) during active forward bending is an aberrant movement pattern observed in patients with low back pain. Increasing load and speed may show different responses in kinematics and motor unit behavior including peak amplitudes (pAMP) and mean firing rates (mFR).<br><br>Objectives<br><br>This study aimed to compare kinematic patterns under different loads and speeds and explored the motor unit behavior in individuals with and without IC.<br><br>Methods<br><br>17 participants were classified as having IC and 10 participants were classified as having no IC from clinical observations. Inertial measurement units were used to quantify kinematic parameters, and decomposition electromyography (dEMG) was used to investigate motor unit behavior. Participants performed 2 sets of 1-minute forward bending under low load and low speed (LL), high load and low speed (HL), and low load and high speed (LH) conditions.<br><br>Results<br><br>Significant between-group differences (P<0.05) were found in kinematic parameters. Significant within-group changes (P<0.05) were found between the LL and HL conditions for all kinematic parameters in individuals with IC. Individuals without IC demonstrated significant within-group changes (P<0.05) between LL and LH in mFR, while individuals without IC showed changes in both pAMP and mFR.<br><br>Conclusion<br><br>These kinematic parameters may represent IC. Changes in motor unit behavior suggest that individuals with and without IC used different strategies to perform this task. Clinicians may consider varying the speed of movement to challenge the trunk neuromuscular control system and design interventions to address motor unit firing rate.
Order of Authors:	Peemongkon Wattananon, PhD
	Sasithorn Kongoun, PhD Candidate
	Katayan Klahan, MSc

Powered by Editorial Manager® and ProduXion Manager® from Aries Systems Corporation

	Sheri P. Silfies, PhD
	John R. Gilliam, PhD
	Jim Richards, PhD
**Additional Information:**	
**Question**	**Response**
**Financial Disclosure**<br><br>Enter a financial disclosure statement that describes the sources of funding for the work included in this submission. Review the submission guidelines for detailed requirements. View published research articles from *PLOS ONE* for specific examples.<br><br>This statement is required for submission and **will appear in the published article** if the submission is accepted. Please make sure it is accurate.<br><br>**Funded studies**<br>Enter a statement with the following details:<br>• Initials of the authors who received each award<br>• Grant numbers awarded to each author<br>• The full name of each funder<br>• URL of each funder website<br>• Did the sponsors or funders play any role in the study design, data collection and analysis, decision to publish, or preparation of the manuscript?<br><br>Did you receive funding for this work?	Yes
Please add funding details.<br>as follow-up to "**Financial Disclosure**<br><br>Enter a financial disclosure statement that describes the sources of funding for the work included in this submission. Review the submission guidelines for detailed requirements. View published research articles from *PLOS ONE* for specific examples.<br><br>This statement is required for submission and **will appear in the published article** if	Initials: PW<br>Grant number: N42A650360<br>Funder: National Research Council of Thailand<br>The funder did not play any role in the study design, data collection and analysis, decision to publish, or preparation of the manuscript

Powered by Editorial Manager® and ProduXion Manager® from Aries Systems Corporation

the submission is accepted. Please make sure it is accurate.<br><br>**Funded studies**<br>Enter a statement with the following details:<br>• Initials of the authors who received each award<br>• Grant numbers awarded to each author<br>• The full name of each funder<br>• URL of each funder website<br>• Did the sponsors or funders play any role in the study design, data collection and analysis, decision to publish, or preparation of the manuscript?<br><br>Did you receive funding for this work?"	
Please select the country of your main research funder (please select carefully as in some cases this is used in fee calculation).<br>as follow-up to "**Financial Disclosure**<br><br>Enter a financial disclosure statement that describes the sources of funding for the work included in this submission. Review the submission guidelines for detailed requirements. View published research articles from *PLOS ONE* for specific examples.<br><br>This statement is required for submission and **will appear in the published article** if the submission is accepted. Please make sure it is accurate.<br><br>**Funded studies**<br>Enter a statement with the following details:<br>• Initials of the authors who received each award<br>• Grant numbers awarded to each author<br>• The full name of each funder<br>• URL of each funder website<br>• Did the sponsors or funders play any role in the study design, data collection and analysis, decision to publish, or preparation of the manuscript?	THAILAND - TH

Did you receive funding for this work?"	
**Competing Interests**<br><br>Use the instructions below to enter a competing interest statement for this submission. On behalf of all authors, disclose any competing interests that could be perceived to bias this work—acknowledging all financial support and any other relevant financial or non-financial competing interests.<br><br>This statement is required for submission and **will appear in the published article** if the submission is accepted. Please make sure it is accurate and that any funding sources listed in your Funding Information later in the submission form are also declared in your Financial Disclosure statement.<br><br>View published research articles from *PLOS ONE* for specific examples.<br><br>**NO authors have competing interests**<br><br>Enter: *The authors have declared that no competing interests exist.*<br><br>**Authors with competing interests**<br><br>Enter competing interest details beginning with this statement:<br><br>*I have read the journal's policy and the authors of this manuscript have the following competing interests: [insert competing interests here]*<br><br>* typeset	The authors have declared that no competing interests exist.
**Ethics Statement**<br><br>Enter an ethics statement for this submission. This statement is required if the study involved:<br><br>• Human participants<br>• Human specimens or tissue	Human participants<br>This study was approved by the Institutional Review Board of Mahidol University (COA No. 2022/118.0711).<br>All participants provided written informed consent prior to participation.

• Vertebrate animals or cephalopods<br>• Vertebrate embryos or tissues<br>• Field research<br><br>Write "N/A" if the submission does not require an ethics statement.<br><br>General guidance is provided below. Consult the submission guidelines for detailed instructions. **Make sure that all information entered here is included in the Methods section of the manuscript.**	

Powered by Editorial Manager® and ProduXion Manager® from Aries Systems Corporation

**Format for specific study types**<br><br>**Human Subject Research (involving human participants and/or tissue)**<br>• Give the name of the institutional review board or ethics committee that approved the study<br>• Include the approval number and/or a statement indicating approval of this research<br>• Indicate the form of consent obtained (written/oral) or the reason that consent was not obtained (e.g. the data were analyzed anonymously)<br><br>**Animal Research (involving vertebrate animals, embryos or tissues)**<br>• Provide the name of the Institutional Animal Care and Use Committee (IACUC) or other relevant ethics board that reviewed the study protocol, and indicate whether they approved this research or granted a formal waiver of ethical approval<br>• Include an approval number if one was obtained<br>• If the study involved *non-human primates*, add *additional details* about animal welfare and steps taken to ameliorate suffering<br>• If anesthesia, euthanasia, or any kind of animal sacrifice is part of the study, include briefly which substances and/or methods were applied<br><br>**Field Research**<br><br>Include the following details if this study involves the collection of plant, animal, or other materials from a natural setting:<br>• Field permit number<br>• Name of the institution or relevant body that granted permission	
**Data Availability**<br><br>Authors are required to make all data underlying the findings described fully available, without restriction, and from the time of publication. PLOS allows rare exceptions to address legal and ethical concerns. See the PLOS Data Policy and FAQ for detailed information.	No - some restrictions will apply

A Data Availability Statement describing where the data can be found is required at submission. Your answers to this question constitute the Data Availability Statement and **will be published in the article**, if accepted.<br><br>**Important:** Stating 'data available on request from the author' is not sufficient. If your data are only available upon request, select 'No' for the first question and explain your exceptional situation in the text box.<br><br>Do the authors confirm that all data underlying the findings described in their manuscript are fully available without restriction?	
**Describe where the data may be found in full sentences. If you are copying our sample text, replace any instances of XXX with the appropriate details.**<br><br>• If the data are **held or will be held in a public repository**, include URLs, accession numbers or DOIs. If this information will only be available after acceptance, indicate this by ticking the box below. For example: *All XXX files are available from the XXX database (accession number(s) XXX, XXX.).*<br>• If the data are all contained **within the manuscript and/or Supporting Information files**, enter the following: *All relevant data are within the manuscript and its Supporting Information files.*<br>• If neither of these applies but you are able to provide **details of access elsewhere**, with or without limitations, please do so. For example:<br><br>*Data cannot be shared publicly because of [XXX]. Data are available from the XXX Institutional Data Access / Ethics Committee (contact via XXX) for researchers who meet the criteria for access to confidential data.*<br><br>*The data underlying the results presented in the study are available from (include the name of the third party*	All relevant data are within the manuscript and its Supporting Information files

and contact information or URL).<br>• This text is appropriate if the data are owned by a third party and authors do not have permission to share the data.<br><br>* typeset	
Additional data availability information:	

Powered by Editorial Manager® and ProduXion Manager® from Aries Systems Corporation

Manuscript

Click here to access/download;Manuscript;Manuscript_dEMG_PLOS

Trunk kinematics and motor unit behavior during different loads and speeds in individuals with and without aberrant movement patterns during active forward bending: A cross-sectional study

Peemongkon Wattananon[1,*], Sasithorn Kongoun[1], Katayan Klahan[1], Sheri P. Silfies[2], John R. Gilliam[2], Jim Richards[3]

[1] Spine Biomechanics Lab, Faculty of Physical Therapy, Mahidol University

[2] Applied Neuromechanics Lab, Arnold School of Public Health, University of South Carolina

[3] Allied Health Research Unit, University of Central Lancashire

This study was funded by National Research Council of Thailand (N42A650360), Mahidol University (MU Talent program), and the Faculty of Physical Therapy, Mahidol University (Research Assistant Fund).

This study was approved by the Institutional Review Board of Mahidol University (COA No. 2022/118.0711).

Address correspondence to:

Peemongkon Wattananon, PT, PhD.

Mail stop: Spine Biomechanics Laboratory, Faculty of Physical Therapy, Mahidol University.

999 Phuttamonthon 4 Road, Salaya, Nakhon Pathom 73170 Thailand.

Email: peemongkon.wat@mahidol.ac.th Tel: 66 2441 5450 Ext. 21803

Acknowledgement

We would like to thank Mahidol University for providing travel grant (Academic and staff mobility program). We also would like to thank the Spine Biomechanics Laboratory, Mahidol University, for providing data collection space and equipment. We also would like to express our appreciation to all participants who joined in this study.

Author contributions

PW has significantly contributed to the conception, research design, data collection, data analysis, and drafting the manuscript. SK, and KK have significantly contributed to research design and data collection. SPS and JRG has substantially contributed to data analysis, drafting, editing, and revising the manuscript. JR has substantially contributed to the data analysis, editing, and revising the manuscript.

Abstract

Background: Instability catch (IC) during active forward bending is an aberrant movement pattern observed in patients with low back pain. Increasing load and speed may show different responses in kinematics and motor unit behavior including peak amplitudes (pAMP) and mean firing rates (mFR).

Objectives: This study aimed to compare kinematic patterns under different loads and speeds and explored the motor unit behavior in individuals with and without IC.

Methods: 17 participants were classified as having IC and 10 participants were classified as having no IC from clinical observations. Inertial measurement units were used to quantify kinematic parameters, and decomposition electromyography (dEMG) was used to investigate motor unit behavior. Participants performed 2 sets of 1-minute forward bending under low load and low speed (LL), high load and low speed (HL), and low load and high speed (LH) conditions.

Results: Significant between-group differences (P<0.05) were found in kinematic parameters. Significant within-group changes (P<0.05) were found between the LL and HL conditions for all kinematic parameters in individuals with IC. Individuals without IC demonstrated significant within-group changes (P<0.05) between LL and LH in mFR, while individuals without IC showed changes in both pAMP and mFR.

Conclusion: These kinematic parameters may represent IC. Changes in motor unit behavior suggest that individuals with and without IC used different strategies to perform this task. Clinicians may consider varying the speed of movement to challenge the trunk neuromuscular control system and design interventions to address motor unit firing rate.

Keywords: Decomposition electromyography, Instability catch, Kinematics, Motor unit behavior, Aberrant movement pattern, Neuromuscular control

1. Introduction

Clinical observation of movement control and coordination during active forward bending of the trunk is one critical part of the physical examination for patients with low back pain (LBP) [1-3]. Aberrant movement patterns during active forward bending cause shear forces and suboptimal tissue loading at the spine resulting in an increased risk of tissue damage [1, 4, 5]. Aberrant movement patterns have consistently been identified in individuals with a history of LBP, and investigators have speculated that this could be due to unresolved lumbar multifidus (LM) muscle dysfunction [1, 4, 6]. Evidence demonstrates the LM does not show spontaneous recovery after an episode of LBP [7-9], with previous work demonstrating a persistent impairment of LM activation using ultrasound imaging in individuals who were in remission following an episode of LBP [8]. Impaired LM activation could compromise lumbar stability and result in an instability catch (IC) during active forward bending. Instability catch (IC) is defined as a momentary quiver, vibration, or shake seen in the lumbar region [1].

Kinematic studies using a dynamic systems approach have characterized IC as a sudden deceleration and acceleration represented by the frequency of local minimum occurrences [10, 11]. However, observed IC in the clinical setting could be triggered by the amplitude of shaking and/or the duration of sudden deceleration and acceleration. Therefore, including kinematic parameters of amplitude and time during deceleration and acceleration may further characterize IC which should result in better representation of this construct. Peak-to-peak (amplitude) and area (amplitude versus time) measures are viable candidates for kinematic parameters to represent IC identified by clinical observation. In addition, changes in the load and speed of the active forward bend may cause changes in neuromuscular demands and result in further changes in the movement patterns [12, 13]. Changes in movement patterns, especially IC, might be caused by motor unit

recruitment patterns. However, evidence to support motor unit behavior underlying IC is still limited.

Typically, healthy individuals initially recruit smaller motor units [14]. As neuromuscular demands increase such as increases in speed, so do motor unit firing rates [15]. If demands increase past the capacity of smaller motor units, additional larger motor units are recruited to match the task requirements [14, 15]. Decomposition electromyography (dEMG) is a new technology that can be used to investigate motor unit behavior by decomposing the EMG signal into individual motor unit action potential trains from which peak amplitude (pAMP) and mean firing rates (mFR) maybe extracted [16-18].

Therefore, this study aimed to compare kinematic patterns during active forward bending including number of acceleration zero-crossings (NUM), peak-to-peak acceleration amplitude (P2P) and area under acceleration graphs (AUC) under different load and speed conditions. Further, we aimed to explore the motor unit behavior (pAMP and mFR) underlying aberrant movement. We hypothesized that increased load and speed would require greater motor unit recruitment resulting in less aberrant movement.

2. Methods

2.1 Study design

This was a cross-sectional study using a two-factor mixed model design to determine the effects of speed and load on trunk kinematics and LM motor unit behavior between people with and without aberrant movement patterns. This study was approved by the University Institutional Review Board (COA No. 2022/118.0711). This study followed the principles of the Declaration of Helsinki. Informed consent for publication of identifying information/images in an online open-

access publication has also been obtained. Data were collected from December 2022 to November 2023.

2.2 Participants

A convenience sample of asymptomatic participants were recruited from the University and surrounding areas. Inclusion criteria were age between 20-40 years, and currently symptom free. Prior episode of LBP was not an exclusion criterion. Participants were excluded if they had definitive neurologic signs including weakness or numbness in the lower extremity, previous spinal surgery, diagnosed osteoporosis, spinal stenosis, inflammatory joint disease, or systemic disease, and a BMI greater than 30 kg/m^2. All participants provided written informed consent before data collection. This sample was part of an ongoing study aimed to explore the potential effects of different interventions on motor unit behavior. A previous study found differences in the number of sudden deceleration and acceleration (zero-crossings) representing IC (judder) between individuals with IC (11.7±4.6 occurrences) and without IC (6.7±2.5 occurrences) [11]. These data were used to determine the sample size required using an 80% power and an alpha of 5%. A total of 20 participants, at least 10 participants per group, were determined necessary to detect differences between groups.

2.3 Instruments and measures

Two Inertial Measurement Unit (IMU) sensors (Trigno Avanti, Delsys Inc., MA, USA) were attached to the lumbar (L1) and sacral (S2) spinous processes to collect kinematic data during the active forward bend movement at 370 Hz, which has been previously used to explore lumbopelvic movements [19, 20], and has been shown to have excellent test-retest reliability to assess movement pattern consistency (coefficient of multiple determination = 0.85) [20].

Two dEMG sensors (Trigno Galileo, Delsys Inc., MA, USA) were attached bilaterally to the lumbar multifidus (2 cm lateral to L4 spinous process) with the reference attached over the iliac crests. These each collected four channels of EMG data from 4 protruding blunted pins with 5-mm inter-pin space. This system has been previously utilized to explore motor unit behavior of back muscles in healthy individuals [21, 22], and the effect of increases in neuromuscular demand during dynamic movements [15].

2.4 Procedure

Demographic data including age, sex, weight, height, and BMI were recorded. Participants were asked to perform 3 repetitions of active forward bending at their most comfortable pace, while a researcher observed their movement and rated the presence or absence of an aberrant movement pattern. Movement pattern observation was performed by a physical therapist with 10 years of clinical experience in assessment and treatment of LBP who did not participate in the data analysis. Participants were rated as having IC if they had obvious shaking in their lumbar area during forward bending. These ratings were used to classify participants to one of two groups with either the presence or absence of IC with pilot data demonstrating a moderate inter-rater reliability of clinical classification (kappa = 0.52).

After classification participants were asked to expose their lumbopelvic area (L1 to S2). Skin preparation was performed using a 70% alcohol swab prior to IMU and dEMG sensor attachment (Figure 1A). Data collection was performed using EMGworks 4.7.3 (Delsys Inc., MA, USA) by two researchers who were blinded to the group assignment. Participants were asked to relax in the prone position and baseline noise was assessed to ensure the value was less than 10 microvolts for dEMG data. If this value was exceeded, the skin preparation procedure was repeated.

Two speeds and two loads were considered. The two speeds were controlled by asking the participants to keep in time with a metronome set at 30 and 50 beats per minute for the downward and upward movements, thus giving a complete movement rate of 15 and 25 repetitions a minute, which has been previously used to assess motor unit behavior and kinematics around the knee [15], and also approximated to the mean velocity of participants performing the movement at a self-selected comfortable pace and the maximum pace that participants could consistently keep in time. Two loads 5% and 10% of body weight were used during the two speeds using kettle bells held in front of the body with arms straight. The 5% of body weight load represented tasks of daily living, and the 10% of body weight load represented the maximum weight that participants could perform for 1 min of repeated forward bending without fatigue. The combination of speed and load were used to create the conditions; low speed and low load (LL), low speed and high load (LH), and high speed and low load (HL). We did not include a high speed and high load condition because participants were unable to consistently perform forward bend throughout 1-minute task in our pilot study. Therefore, we included only LL, LH and HL conditions for this study. The participants were asked to perform 2 sets of forward bends to 45-degrees of lumbar flexion, which was standardized by adjusting the height of a target bar (Figure 1B and 1C), each for 1 minute with a 5-minute rest between sets. The three conditions (LL, LH, and HL) were performed in a random sequence. We did not include high speed and high load in our study because our pilot work found that most participants reported muscle fatigue after completing this condition. IMU and dEMG data were simultaneously collected during each condition.

2.5 Data reduction

Kinematic data reduction was performed using a custom LabVIEW program (National Instruments, TX, USA). All IMU data were filtered using a second order lowpass Butterworth

filter at 20 Hz. Assessments of sagittal plane aberrant movements during active forward bending were evaluated from the flexion/extension angular velocity data from the lumbar sensor. Start and stop events (neutral position to targeted position) were identified using 5% of maximum lumbar angular velocity as a cut-off point. Mean angular velocity (MV), peak angular velocity (PV), as well as number of acceleration zero-crossings (NUM), peak-to-peak acceleration amplitude (P2P), and total area under sudden deceleration and acceleration curves (AUC) were derived. These parameters were used for further statistical analysis.

dEMG data processing was performed using the Neuromap software (Delsys Inc., MA, USA). This program uses algorithms to identify individual action potentials in the dEMG signal through artificial intelligence techniques which decompose the superposed signal to individual motor unit action potential trains [16-18]. The peak motor unit amplitude (pAMP) and mean firing rates (mFR) were calculated.

2.6 Statistical analysis

Statistical analysis was performed using SPSS version 21 (IBM Corp., NY, USA). An independent t-test was used to determine the difference in age, BMI, mean angular velocity (for each condition), while a chi-square test was performed to determine the difference in sex proportion between groups.

The distribution of data was tested using Shapiro-Wilk tests, and all kinematic and dEMG data were normally distributed. Before main data analysis, we used data from 2 sets of 1-minute forward bends to calculate test-retest reliability and standard error of measurement (SEM) for each parameter to strengthen our internal validity. We further used the SEM to determine whether within and between-group differences were resulted from measurement error or not. We used a two-factor mixed model ANOVA with post-hoc least significant difference (LSD) and SEM to determine

interaction and main effects of group (presence and absence of IC) and condition (LL, LH and HL).

In addition, dEMG (pAMP and mFR) data from individual participants were used to construct profiles indicating increased or decreased response to changes in speed and load in each parameter using LL as the reference value. The frequency and percentage of participants who demonstrated an increase from their reference dEMG parameters or decrease from their kinematic parameters in response to increased speed or load was calculated for each parameter. These percentages were then used to characterize potential neuromuscular strategies for individuals with and without IC.

3. Results

Twenty-seven individuals were recruited who were classified as either having IC or not during active forward bending. Ten individuals were classified as having no IC (mean age 24.0 years, 4 females, and BMI 23.3 kg/m^2) and 17 individuals were classified as having IC (mean age 22.4 years, 10 females, and BMI 21.4 kg/m^2). Demographic and MV data are presented in Table 1. No significant differences were found between individuals with and without IC for age, sex, BMI, or MV.

For dEMG parameters, data from one participant could not be decomposed due to a technical problem, this individual was excluded from the statistical analysis. Our dataset also demonstrated no significant differences in dEMG parameters between left and right sides; therefore, we used side-to-side averaged values for statistical analysis. Although sex could potentially affect dEMG parameters, we did not find any interactions between sex and other parameters in our dataset which was consistent with another dEMG study [15].

Test-test reliability for kinematic parameters (NUM, P2P, and AUC) showed moderate to excellent ($ICC_{2,k}$ = 0.95, 0.72, and 0.91), respectively. SEM values were 0.7 occurrences, 0.35 deg/sec, and 6.03 units, respectively. Test-test reliability for dEMG parameters (pAMP and mFR) were uniformly excellent ($ICC_{2,k}$ = 0.96 and 0.93, respectively). SEM values were 8.12 microvolts, and 0.37 pps, respectively.

Table 2 demonstrates the means, standard errors, and effect sizes for interaction (Group×Condition) and main effect of group and condition for kinematics and motor unit behavior. No significant interactions (P>0.05) were seen between group and condition, but significant main effects of condition (P<0.05) with large effect sizes were seen for all parameters, except pAMP. In addition, significant main effects of group (P<0.05) with large effect sizes were seen for P2P and AUC, while there was a trend (P=0.053) with large effect size for NUM.

Table 3 illustrates pairwise comparisons between groups and conditions for all kinematic and dEMG parameters. Post-hoc pairwise comparisons demonstrated significant between-group differences (P<0.05) and exceeding the SEM in kinematic parameters, except for NUM, in LL and HL conditions (P<0.05). However, the non-significant difference in Num between groups in both conditions exceeded the SEM. Within-group comparisons did not show significant within-group changes (P>0.05) between LL and LH in both groups for all kinematic parameters. We found significant changes (P<0.05) comparing LL and HL conditions in both groups for Num, but in IC group alone for P2P and AUC. In addition, changes in NUM and AUC exceeded the SEM, while changes in P2P did not exceed the SEM either group.

Although the results showed non-significant differences in pAMP, we wanted to further explore the motor unit behavior by performing comparisons using LSD (Table 3). No significant within-group changes (P>0.05) were seen between LL and LH for pAMP and mFR in either group.

When comparing LL and HL, the IC group demonstrated significant changes ($P<0.05$) beyond the SEM in both pAMP and mFR. The group without IC findings for aberrant movement showed a significant change ($P<0.05$) exceeding the SEM only in mFR. Table 4 presents a summary of individual profiles and potential strategies based on changes in motor unit behavior in response to each condition for each individual.

4. Discussion

Overall, kinematic results demonstrated individuals with IC had greater NUM, P2P and AUC than those without IC suggesting these parameters are representative of the presence of this aberrant movement pattern. Although the IC group did not show significantly greater NUM in the LL and HL conditions, this group still shows a trend toward greater NUM and the difference between groups exceeded the SEM. Our results were consistent with a previous study using kinematics that found a greater number of local minimum occurrences (comparable to NUM in our study) in individuals with aberrant movement [11]. Similar to the present findings, they found a mean difference between those with typical movement and IC was 2.4.

A potential limitation of measuring NUM alone is that it represents only the number of sudden decelerations and accelerations without consideration for the amplitude and timing of IC. These movement qualities can be quantified by P2P (taking amplitude into consideration) and AUC (taking amplitude and time into consideration). The same limitation measuring NUM may also be present with clinical observation of active forward bending in which small shaking (high NUM, but low amplitude) may not be detectable to the naked eye. Under the condition of increased load (LH), neither group showed significant changes in kinematic variables compared with the reference level. However, individuals with clinically observed IC had greater NUM, P2P, and AUC compared with those without IC. Additionally, effect sizes for group differences were amplified

under the higher load condition. This may be the result of a greater challenge for the neuromuscular system [14]. Future investigations aiming to compare differences in kinematics in this population, might consider loaded conditions in order to elicit differences in task performance.

Within-group comparisons between LL and LH conditions indicate that increasing load did not impact movement patterns. Alternatively, significant decreases, larger than the SEM, were observed for NUM and AUC when speed was increased (HL); P2P also had a significant reduction in the IC group with increasing speed, but changes were within measurement error. This suggest changes in AUC could be due to changes in NUM, if P2P remained the same. Although the group without clinical signs of aberrant movement demonstrated a reduction trend, greater than the SEM, in AUC when increasing speed, these features might not be observed by clinicians because these values were less than baseline (LL condition). Considering between group and between condition differences, it appears that AUC is the most sensitive kinematic measure.

We did not find any changes in motor unit behavior when adding load. This is consistent with our findings from kinematics. However, changes were observed when increasing speed. These findings suggest that participants with and without IC used different strategies to perform the task where individuals without IC increased mFR, while individuals with IC increased pAMP and mFR. Theoretically, greater pAMP could be related to the recruitment of larger motor units [14, 23]. Although these larger motor units can generate greater force, it is easily fatigable [23]. This could increase the risk of injury in situations that require repetitive and prolonged performance of a task. Motor unit behavior parameters are intercorrelated [14, 17], therefore, we characterized individual responses to explore potential strategies for each group and investigated the effects of a history of LBP on these parameters (Table 3).

Differences in movement strategies between groups could be responsible for non-significant between-group differences when considering each motor unit behavior feature separately. This is evident only by exploring individual profiles. Individual subject profiles revealed that the IC group had a greater percentage of individuals with a history of LBP (64.7%) compared to those without IC (30%). Although they were asymptomatic at the time of testing, participants with a history of LBP continued to present with aberrant movement. This finding is consistent with previous reports where aberrant movements were observed after the resolution of pain or following an episode of LBP [1, 6]. Aberrant movements can cause shear forces and suboptimal tissue loading thereby increasing the risk of re-injury in those with a history of LBP [4, 5]. According to our motor unit behavior findings, it seems that these aberrant movement could be caused by altered motor unit behavior. Approximately twenty-two percent of individuals without IC increased pAMP and mFR as a strategy to perform forward bend with higher load, while 23.5% of the IC group increased mFR during this condition. When increasing speed, a majority (55.6%) of individuals without IC increased mFR as a strategy, which is consistent with the results presented in Table 3. In contrast, a greater percentage (35.3%) of individuals with IC relied on increased pAMP to accommodate faster forward bending. Motor unit firing rate strategy in individuals without IC were consistent with strategies commonly reported in dEMG studies [15, 24]. This finding suggests that when individuals are required to perform faster movements, the neuromuscular control system increases motor unit firing rate to match the demand being placed on the neuromuscular control system [15].

The IC group demonstrated a smaller percentage of individuals who increased mFR in response to the condition with increased speed. Some individuals with IC (dEMG08 and 17) utilized motor unit behavior strategies that were sufficient to improve movement control as

evidenced by decreased AUC during this condition, while other participants in the IC group (dEMG 03, 14, 24, 06, 13, 18) demonstrate changes in motor unit behavior, but these strategies were insufficient to impact AUC. Additionally, participants 9, 10, 11, 15, 22 did not demonstrate any changes in motor unit behavior variables, while decreasing AUC during this condition. It is worth noting that four of these participants had no history of LBP, which may have been sufficient to improve movement control. It is also important to consider that active forward bending requires significant contribution from muscle groups such as the erector spinae and gluteal muscles, in addition to the LM. Evidence supports altered muscle activation patterns in these muscle groups in patients with LBP [3, 23, 25]. Future studies should include a wider array of trunk and hip muscles to better understanding motor unit behaviors in this population.

Although statistical analysis found a non-significant between-group difference in mean velocity across conditions, individuals with IC group moved slightly faster than those without IC. This might suggest the inability to control the movement during forward bend. To the best of our knowledge, this is the first study to investigate motor unit behavior response to conditions of increased load and speed. Therefore, we are unable to compare our results with other studies. However, one study has reported on the motor unit behavior of females analyzing dominant and non-dominant back muscles [21]. They reported greater mFR than our findings. The different values could reflect differences in protocols. They collected data from the lumbar erector spinae using a 40% bodyweight load which resulted in a greater neuromuscular requirement. Additionally, the lumbar erector spinae primarily function as a force generator for trunk movement, which should be composed of larger motor units and the ability to generate force at higher mFR [26].

Our study has some limitations. First, clinical observation and kinematics were collected during the eccentric phase of movement, while dEMG considered both eccentric and concentric

contractions. Our study investigated only the LM muscles; therefore, the motor unit behavior changes in our study are not generalizable to other muscles. Future studies should include other muscles involved in this forward bend task. We aimed to explore motor unit behavior in response to increased load and speed. Our primary statistical analysis did not show significant findings; however, our detailed exploration of group and individual movement profiles should inform the design future studies. Replication of this study is required.

5. Conclusion

Kinematic results demonstrated individuals with IC had greater NUM, P2P and AUC than those without IC suggesting these parameters can represent IC. Changes in motor unit behavior were found when increasing speed and these changes were different between individuals with and without IC suggesting these groups used a different neuromuscular strategy to perform the task.

Declaration of Competing Interest

The authors declare no conflict of interest.

References

1. Biely SA, Silfies SP, Smith SS, Hicks GE. Clinical observation of standing trunk movements: what do the aberrant movement patterns tell us? *J Orthop Sports Phys Ther*. 2014;44(4):262-72.
2. Laird RA, Gilbert J, Kent P, Keating JL. Comparing lumbo-pelvic kinematics in people with and without back pain: a systematic review and meta-analysis. *BMC Musculoskelet Disord*. 10 2014;15:229.
3. Wattananon P, Sinsurin K, Somprasong S. Association between lumbopelvic motion and muscle activation in patients with non-specific low back pain during forward bending task: A cross-sectional study. *Hong Kong Physiother J*. 2020;40(1):29-37.
4. MacDonald D, Moseley GL, Hodges PW. Why do some patients keep hurting their back? Evidence of ongoing back muscle dysfunction during remission from recurrent back pain. *Pain*. 2009;142(3):183-8.
5. Sahrmann S, Azevedo DC, Dillen LV. Diagnosis and treatment of movement system impairment syndromes. *Braz J Phys Ther*. 2017;21(6):391-399.
6. Wattananon P, Kong-oun S, Chuenpimonchankit P, et al. Clinical prediction rule validity to identify individuals with recurrent low back pain. journal article. *Physiother Q*. 2022;30(1):27-32.
7. Hides JA, Richardson CA, Jull GA. Multifidus muscle recovery is not automatic after resolution of acute, first-episode low back pain. *Spine (Phila Pa 1976)*. 1996;21(23):2763-9.
8. Sungnak P, Songjaroen S, Krityakiarana W, Wang HK, Richards J, Wattananon P. Individuals with impaired lumbopelvic control demonstrate lumbar multifidus muscle activation deficit

using ultrasound imaging in conjunction with electrical stimulation: a cross-sectional study. *Arch Phys Med Rehabil*. 2022;103(10):1951-1957.

9. Thu KW, Maharjan S, Sornkaew K, Kongoun S, Wattananon P. Multifidus muscle contractility deficit was not specific to the painful side in patients with chronic low back pain during remission: a cross-sectional study. *J Pain Res*. 2022;15:1457-1463.
10. Spinelli BA, Wattananon P, Silfies S, Talaty M, Ebaugh D. Using kinematics and a dynamical systems approach to enhance understanding of clinically observed aberrant movement patterns. *Man Ther*. 2015;20(1):221-6.
11. Wattananon P, Ebaugh D, Biely SA, Smith SS, Hicks GE, Silfies SP. Kinematic characterization of clinically observed aberrant movement patterns in patients with non-specific low back pain: a cross-sectional study. *BMC Musculoskelet Disord*. 2017;18(1):455.
12. Asgari M, Sanjari MA, Mokhtarinia HR, Moeini Sedeh S, Khalaf K, Parnianpour M. The effects of movement speed on kinematic variability and dynamic stability of the trunk in healthy individuals and low back pain patients. *Clin Biomech (Bristol, Avon)*. 2015;30(7):682-8.
13. Larivière C, Gagnon D, Loisel P. The effect of load on the coordination of the trunk for subjects with and without chronic low back pain during flexion-extension and lateral bending tasks. *Clin Biomech (Bristol, Avon)*. 2000;15(6):407-16.
14. De Luca CJ. Control properties of motor units. *J Exp Biol*. 1985;115:125-36.
15. Orantes-Gonzalez E, Heredia-Jimenez J, Lindley SB, Richards JD, Chapman GJ. An exploration of the motor unit behaviour during the concentric and eccentric phases of a squat task performed at different speeds. *Sports Biomech*. 2023:1-12.

16. De Luca CJ, Chang SS, Roy SH, Kline JC, Nawab SH. Decomposition of surface EMG signals from cyclic dynamic contractions. *J Neurophysiol*. 2015;113(6):1941-51.
17. De Luca CJ, Contessa P. Biomechanical benefits of the Onion-Skin motor unit control scheme. *J Biomech*. 2015;48(2):195-203.
18. Nawab SH, Chang SS, De Luca CJ. High-yield decomposition of surface EMG signals. *Clin Neurophysiol*. 2010;121(10):1602-15.
19. Budini K, Richards J, Cole T, et al. An exploration of the use of Inertial Measurement Units in the assessment of dynamic postural control of the knee and the effect of bracing and taping. *Physiother Pract Res*. 2018;39:91-98.
20. Wattananon P, Kongoun S, Chohan A, Richards J. The use of statistical parametric mapping to determine altered movement patterns in people with chronic low back pain. *J Biomech*. 2023;153:111601.
21. Silva MF, Dias JM, Pereira LM, et al. Determination of the motor unit behavior of lumbar erector spinae muscles through surface EMG decomposition technology in healthy female subjects. *Muscle Nerve*. 2017;55(1):28-34.
22. Wattananon P, Songjaroen S, Sungnak P, Richards J. Neuromuscular responses to combined neuromuscular electrical stimulation and motor control exercises in a patient with recurrent low back pain: A single subject research report. *Physiother Theory Pract*. 2022:1-6.
23. Hodges PW, Danneels L. Changes in structure and function of the back muscles in low back pain: different time points, observations, and mechanisms. *J Orthop Sports Phys Ther*. 2019;49(6):464-476.
24. Oliveira AS, Negro F. Neural control of matched motor units during muscle shortening and lengthening at increasing velocities. *J Appl Physiol*. 2021;130(6):1798-1813.

25. van Dieen JH, Reeves NP, Kawchuk G, van Dillen LR, Hodges PW. Motor control changes in low back pain: divergence in presentations and mechanisms. *J Orthop Sports Phys Ther*. 2019;49(6):370-379.

26. Agten A, Stevens S, Verbrugghe J, Eijnde BO, Timmermans A, Vandenabeele F. The lumbar multifidus is characterised by larger type I muscle fibres compared to the erector spinae. *Anat Cell Biol*. 2020;53(2):143-150.

Table 1. Demographic data and performance for each condition

Parameter	Negative (n=10) Mean (SD)	Positive (n=17) Mean (SD)
Age (year)	24.0 (4.6)	22.4 (1.3)
Sex (male/female)	6/4	7/10
BMI (kg/m^2)	23.3 (3.4)	21.4 (1.8)
LL_MV (deg/sec)	49.50 (9.32)	57.44 (11.27)
LH_MV (deg/sec)	48.98 (10.39)	55.05 (14.13)
HL_MV (deg/sec)	65.36 (12.08)	72.15 (12.38)

BMI=body mass index; LL=low speed and low load; LH=low speed and high load; HL=high speed and low load; MV=mean angular velocity; SD=standard deviation

Table 2. Mean and standard error for interaction (ABM×Condition) and main effect of group and condition for kinematics and motor unit behavior

Parameter	Interaction (ABM×Condition)		Main effect								
			ABM				Condition				
	p-value	partial η^2	Negative Mean (SD)	Positive Mean (SD)	p-value	partial η^2	LL Mean (SD)	LH Mean (SD)	HL Mean (SD)	p-value	partial η^2
NUM (occurrences)	0.207	0.06	3.6 (0.6)	5.2 (0.5)	0.053	0.14	5.1 (0.5)	5.2 (0.5)	2.9 (0.3)	<0.001*	0.51
P2P (deg/sec)	0.573	0.02	0.57 (0.21)	1.45 (0.16)	0.003*	0.30	1.09 (0.15)	1.05 (0.13)	0.90 (0.14)	0.032*	0.13
AUC	0.074	0.10	11.42 (6.07)	37.11 (4.66)	0.003*	0.31	28.38 (4.88)	26.41 (3.83)	18.01 (3.30)	<0.001*	0.31
pAMP (microvolts)	0.352	0.04	89.43 (13.13)	91.77 (9.55)	0.887	0.01	85.78 (9.77)	91.10 (8.07)	94.91 (8.75)	0.352	0.04
mFR (pps)	0.795	0.01	5.10 (0.31)	4.76 (0.22)	0.374	0.03	4.64 (0.25)	4.79 (0.18)	5.35 (0.21)	<0.001*	0.27

ABM=aberrant movement; NUM=number of zero-crossing; P2P=peak-to-peak of sudden deceleration and acceleration amplitude; AUC=area under sudden deceleration and acceleration curve; pAMP=peak motor unit amplitude; mFR=mean firing rate; LL=low speed low load; LH=low speed high load; HL=high speed low load; partial η^2=effect size partial eta-squared; SD=standard deviation

*=significant difference (p<0.05)

Table 3. Mean and standard deviation for pairwise comparisons between negative and positive groups and among three conditions

Parameter	SEM	ABM	Condition<br>LL<br>Mean (SD)	LH<br>Mean (SD)	HL<br>Mean (SD)	LL vs LH<br>Diff	p-value	ES	LL vs HL<br>Diff	p-value	ES
NUM (occurrences)	0.7	Negative	4.4 (2.1)	4.0 (1.3)	2.4 (1.0)	0.4	0.548	0.25	2.0[a]	0.001*	1.40
		Positive	5.9 (2.9)	6.3 (2.8)	3.4 (1.8)	-0.4	0.301	0.20	2.5[a]	<0.001*	1.36
		Diff	1.5[a]	2.3[a]	1.0[a]						
		p-value	0.172	0.024*	0.105						
		ES	0.59	1.05	0.69						
P2P (deg/sec)	0.35	Negative	0.66 (0.31)	0.57 (0.24)	0.49 (0.27)	0.09	0.505	0.46	0.17	0.095	0.77
		Positive	1.51 (0.94)	1.54 (0.78)	1.31 (0.86)	-0.03	0.804	0.06	0.20	0.014*	0.57
		Diff	0.85[a]	0.97[a]	0.82[a]						
		p-value	0.011*	0.001*	0.007*						
		ES	1.21	1.68	1.29						
AUC	6.03	Negative	14.42 (8.53)	11.55 (5.17)	8.28 (4.87)	2.87	0.511	0.48	6.14[a]	0.111	0.97
		Positive	42.34 (29.95)	41.27 (23.72)	27.73 (20.37)	1.07	0.750	0.07	14.61[a]	<0.001*	1.05
		Diff	27.92[a]	29.72[a]	19.45[a]						
		p-value	0.008*	0.001*	0.007*						
		ES	1.27	1.79	1.31						
pAMP (microvolts)	8.12	Negative	89.81 (71.37)	88.21 (56.68)	90.27 (53.73)	-1.6	0.897	0.03	0.46	0.962	0.04
		Positive	81.74 (28.64)	94.00 (26.37)	99.55 (35.47)	12.26[a]	0.184	0.85	17.81[a]	0.016*	0.78
		Diff	-8.07	5.79	9.28[a]						
		p-value	0.683	0.723	0.601						
		ES	0.15	0.13	0.20						
mFR (pps)	0.37	Negative	4.84 (1.66)	4.89 (0.98)	5.57 (1.09)	0.05	0.872	0.04	0.73[a]	0.021*	0.62
		Positive	4.45 (0.93)	4.69 (0.81)	5.14 (0.96)	0.24	0.323	0.33	0.69[a]	0.011*	0.98
		Diff	-0.39[a]	0.20	0.43[a]						
		p-value	0.443	0.573	0.310						
		ES	0.29	0.22	0.42						

ABM=aberrant movement; NUM=number of zero-crossing; P2P=peak-to-peak of sudden deceleration and acceleration amplitude; AUC=area under sudden deceleration and acceleration curve; pAMP=peak motor unit amplitude; mFR=mean firing rate; SEM=Standard error of measurement; LL=low speed low load; LH=low speed high load; HL=high speed low load; SD=standard deviation; ES=effect size (Cohen's d).
*=significant difference (p<0.05); [a]=difference exceeds SEM

Table 4. Summary of individual profiles and potential strategies

Participant	Sex	HxLBP	Load: Motor unit behavior		Load: Kinematics	Speed: Motor unit behavior		Speed: Kinematics
			pAMP	mFR	AUC	pAMP	mFR	AUC
No Observed Instability Catch								
dEMG01	M	Y	↓	↔	↔	↓	↑	↔
dEMG04	M	N	↔	↔	↔	↔	↔	↔
dEMG12	M	N	↑	↓	↔	↔	↔	↓
dEMG16	M	N	↑	↔	↔	↑	↑	↔
dEMG21	M	Y	↔	↔	↔	↔	↔	↔
dEMG27	M	N	↔	↔	↔	↔	↔	↔
dEMG07	F	N	N/A	N/A	↔	N/A	N/A	↔
dEMG19	F	Y	↔	↑	↔	↔	↑	↔
dEMG23	F	N	↔	↔	↔	↔	↑	↔
dEMG26	F	N	↓	↑	↔	↔	↑	↔
Sum	4	3	2	2	0	1	5	1
Percentage	40.0%F	30.0	22.2[a]	22.2[a]	0	11.1[a]	55.6[a]	14.3
Observed Instability Catch								
dEMG03	M	N	↑	↔	↑	↑	↔	↔
dEMG08	M	Y	↔	↔	↔	↑	↔	↓
dEMG09	M	N	↔	↔	↔	↔	↔	↓
dEMG10	M	N	↔	↔	↔	↔	↔	↓
dEMG11	M	N	↔	↑	↓	↔	↔	↓
dEMG14	M	Y	↔	↔	↑	↑	↑	↔
dEMG24	M	Y	↔	↑	↔	↔	↑	↔
dEMG02	F	Y	↔	↔	↔	↔	↔	↔
dEMG05	F	N	↑	↑	↔	↔	↔	↔
dEMG06	F	Y	↔	↔	↔	↑	↔	↔
dEMG13	F	Y	↔	↔	↓	↑	↑	↔
dEMG15	F	N	↔	↔	↔	↔	↔	↓
dEMG17	F	Y	↔	↑	↔	↔	↑	↓
dEMG18	F	Y	↔	↔	↔	↑	↔	↔
dEMG20	F	Y	↔	↔	↔	↔	↔	↔
dEMG22	F	Y	↔	↔	↓	↔	↔	↓
dEMG25	F	Y	↔	↔	↔	↔	↔	↔
Sum	10	11	2	4	3	6	4	7
Percentage	58.8%F	64.7	11.8	23.5	17.6	35.3	23.5	41.2

M=male; F=female; HxLBP= history of low back pain; pAMP=peak motor unit amplitude; mFR=mean firing rate; AUC=area under sudden deceleration and acceleration curve; [a]=based on 9 participants; Note: only increases were counted for dEMG parameters, and decreases were counted for kinematics parameter

Figure legend

Figure 1. Inertial measurement unit (IMU) and decomposition electromyography (dEMG) sensor locations (A) and task starting (B) and end (C) positions.

Figure 1

Click here to access/download;Figure;Figure_1.tiff

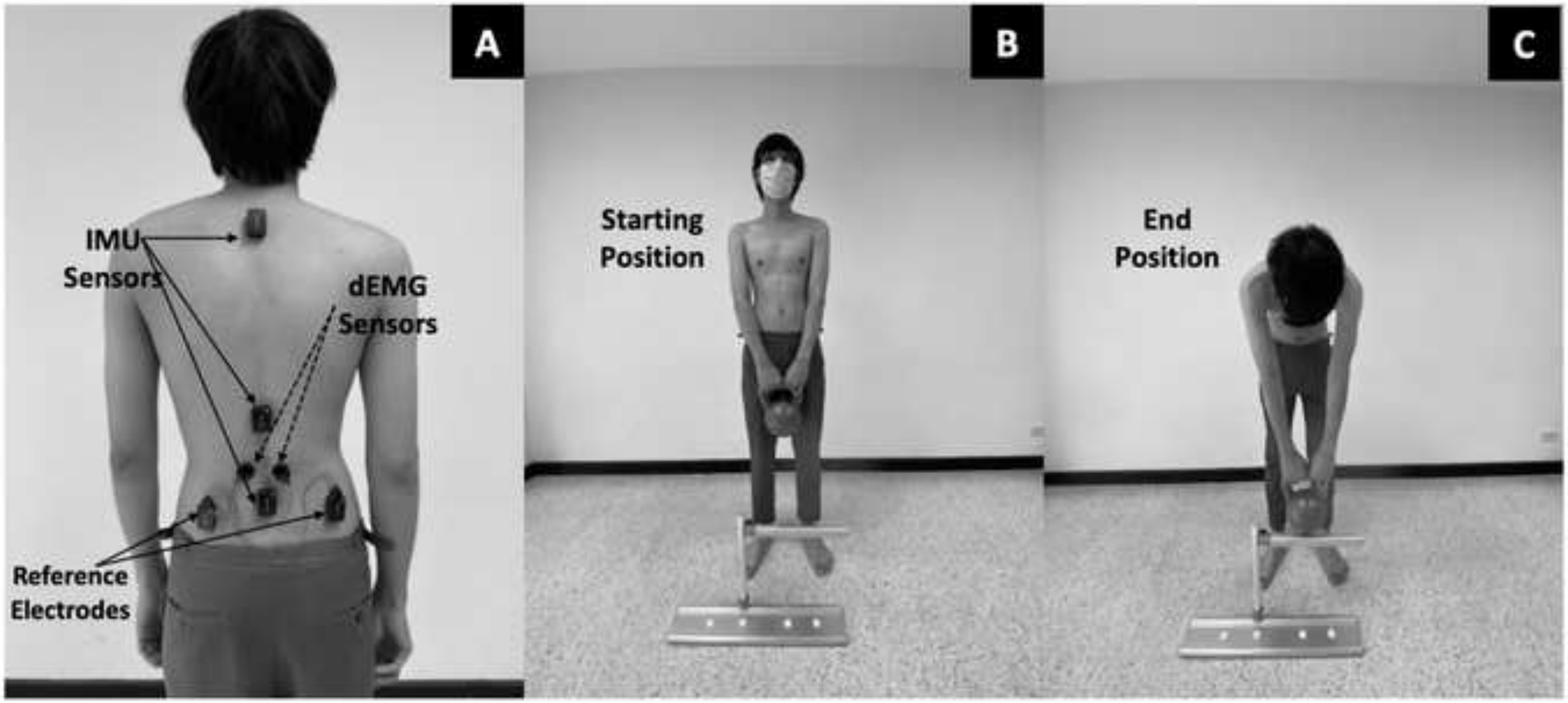

Supplement: Attachment — Submitted filename: PONE-D-24-57098_reviewer_reviewers comments.pdf [file pone.0321084.s002.pdf]
